# Transversus Abdominis Plane Versus Ilioinguinal and Iliohypogastric Nerve Blocks for Analgesia Following Open Inguinal Herniorrhaphy*

**DOI:** 10.5041/RMMJ.10248

**Published:** 2016-07-28

**Authors:** Anatoli Stav, Leonid Reytman, Michael-Yohay Stav, Anton Troitsa, Mark Kirshon, Ricardo Alfici, Mickey Dudkiewicz, Ahud Sternberg

**Affiliations:** 1 Postanesthesia Care Unit, Hillel Yaffe Medical Center, Hadera, Israel; 2 The Ruth and Bruce Rappaport Faculty of Medicine, Technion–Israel Institute of Technology, Haifa, Israel; 3 Department of Anesthesiology, Hillel Yaffe Medical Center, Hadera, Israel; 4 Department of Surgery A, Hillel Yaffe Medical Center, Hadera, Israel; 5 Department of Surgery B, Hillel Yaffe Medical Center, Hadera, Israel; 6 Director-General, Hillel Yaffe Medical Center, Hadera, Israel

**Keywords:** Analgesia, nerve block, open herniorrhaphy, pain, post-op, ultrasound

## Abstract

**Objectives:**

We hypothesized that preoperative (pre-op) ultrasound (US)-guided posterior transversus abdominis plane block (TAP) and US-guided ilioinguinal and iliohypogastric nerve block (ILI+IHG) will produce a comparable analgesia after Lichtenstein patch tension-free method of open inguinal hernia repair in adult men. The genital branch of the genitofemoral nerve will be blocked separately.

**Methods:**

This is a prospective, randomized, controlled, and observer-blinded clinical study. A total of 166 adult men were randomly assigned to one of three groups: a pre-op TAP group, a pre-op ILI+IHG group, and a control group. An intraoperative block of the genital branch of the genitofemoral nerve was performed in all patients in all three groups, followed by postoperative patient-controlled intravenous analgesia with morphine. The pain intensity and morphine consumption immediately after surgery and during the 24 hours after surgery were compared between the groups.

**Results:**

A total of 149 patients completed the study protocol. The intensity of pain immediately after surgery and morphine consumption were similar in the two “block” groups; however, they were significantly decreased compared with the control group. During the 24 hours after surgery, morphine consumption in the ILI+IHG group decreased compared with the TAP group, as well as in each “block” group versus the control group. Twenty-four hours after surgery, all evaluated parameters were similar.

**Conclusion:**

Ultrasound-guided ILI+IHG provided better pain control than US-guided posterior TAP following the Lichtenstein patch tension-free method of open inguinal hernia repair in men during 24 hours after surgery. (ClinicalTrials.gov number: NCT01429480.)

## INTRODUCTION

The Lichtenstein patch tension-free method of open inguinal hernia repair^1^ is commonly used under general anesthesia with the facilitation of ilioinguinal and iliohypogastric nerve blocks (ILI+IHG) to improve postoperative (post-op) analgesia.^2^

The PROSPECT Working Group (http://www.postoppain.org/working-group/) published a procedure-specific review and guideline for analgesia following inguinal hernia repair in 2012, in which ILI+IHG were strongly recommended preoperatively (pre-op) and during surgery (grade A recommendation based on randomized clinical trials).^3^ According to the new guideline published in February 2016, the use of peripheral regional anesthetic techniques is strongly recommended as a component of multimodal analgesia for pain management following open inguinal hernia repair.^4^

Ultrasound (US)-guided posterior transversus abdominis plane block (TAP) has been described as an appropriate method of post-op analgesia for abdominal wall incisions below the umbilicus. Inguinal hernia repair was also included.^5^–^7^ Aveline et al. compared the efficacy of pre-op US-guided TAP to conventional (landmark-guided technique) ILI+IHG and concluded that better post-op pain relief was achieved following US-guided TAP.^8^ Fredrickson et al. concluded that, in children, US-guided ILI+IHG provides more effective analgesia compared with US-guided TAP following inguinal herniotomy, hydrocelectomy, and orchiopexy.^9^ Wang et al. conducted a meta-analysis to evaluate the clinical efficacy of US-guided ILI+IHG and US-guided TAP for perioperative analgesia in patients undergoing open inguinal surgery; however, only four articles were included in the meta-analysis:^10^ two randomized controlled trials were performed in pediatric patients,^11^,^12^ and two trials were conducted in adults.^8^,^13^ The authors concluded that US-guided ILI+IHG or TAP is associated with improved perioperative analgesia compared with the landmark-based technique.

Anatomically, sensory innervation of the male inguinal region is from the Th12–L2 nerves, and “the spermatic sympathetic plexus contains the sensory fibers for the testis.”^9^ The ilioinguinal and iliohypogastric nerves (branches of Th12 and L1) pass between the internal oblique and transversus abdominis muscles at the level immediately superior to the anterior superior iliac spine. More medial ilioinguinal and iliohypogastric nerves pierce the internal oblique abdominal muscle and lie near the internal inguinal ring between it and the external oblique muscles.^9^ The ilioinguinal and iliohypogastric nerves innervate part of the structures in the inguinal canal and lie on the anterior surface of the spermatic cord.^9^ The genital branch of the genitofemoral nerve (L1, L2) lies on the posterior aspect of the spermatic cord after entering the inguinal canal through the internal inguinal ring;^9^ it provides motor and sensory innervation to the cremaster muscle and anterolateral aspect of the scrotum in men.^14^ Although ILI+IHG has been shown to provide insufficient analgesia, the addition of a genitofemoral nerve block may improve the analgesic effect.^15^ According to the described technique for both evaluated blocks, local anesthetic should be injected into the fascial plane between the internal oblique and transversus abdominis muscles.^5^–^7^,^16^ There is a difference in the volume and anatomical point of injection. The recommended volume of injected local anesthetic for TAP is 20–30 mL, as compared with 10 mL for ILI+IHG.^16^ The point of needle insertion for an US-guided TAP is more proximal compared with US-guided ILI+IHG.^5^–^7^,^16^

We suggest that the ilioinguinal and iliohypogastric nerves may be appropriately blocked using both US-guided techniques, TAP and ILI+IHG, since both techniques produce the same block of the ilioinguinal and iliohypogastric nerves; the only difference is that TAP is a compartment block, while ILI+IHG blocks the truncal.

In order to compare the effect of TAP and ILI+IHG on post-op pain intensity, pain must be eliminated from the structures innervated by the genital branch of the genitofemoral nerve; an intraoperative block of the genital branch of the genitofemoral nerve should be used in all patients; and the same pharmacological treatment should be offered for post-op pain. Our model includes post-op, patient-controlled analgesia with intravenous morphine (PCA). No previous trials have compared US-guided TAP versus US-guided ILI+IHG in adult men undergoing inguinal hernia repair using the open Lichtenstein patch tension-free method. We tested the hypothesis that both US-guided TAP and US-guided ILI+IHG provide comparable post-op analgesia, together with morphine consumption, following Lichtenstein patch hernioplasty during the first 24 hours post-op.

## METHODS

This is a prospective, randomized, controlled, and observer-blinded clinical study; enrollment began after receiving Hillel Yaffe Review Board approval and written informed patient consent. Study eligibility was as follows: male; over the age of 18; physical status of I–III based on the American Society of Anesthesiologists criteria; and scheduled to undergo elective inguinal hernioplasty using the unilateral open Lichtenstein patch tension-free method. The rationale only to include males in the trial was based on the aim to compare data among maximally identical groups. In total, 186 patients were eligible for the study.

Exclusion criteria were recurrent hernia, previous episode of incarceration, inguino-scrotal hernia, or sliding inguinal hernia.^17^ It should be noted that manipulating sliding hernias during an open herniorrhaphy can produce visceral pain, whereas pre- or intraoperative blocks of the somatic peripheral nerves (i.e. TAP, ILI+IHG, and genital branch of the genitofemoral nerve) do not cause visceral pain. Additional exclusion criteria included being under the age of 18, presence of a local skin infection near the block injection site, allergy to local anesthetics, demonstrated opioid dependency, international normalized ratio (INR) greater than 1.4, platelet count less than 100,000, chronic pain, dementia, or an inability to comprehend the pain scale or use the PCA device.

Patients were instructed regarding the use of a 100 mm visual analogue scale (VAS) graded from 0 (without pain) to 100 (intolerable pain).

**Figure 1:**
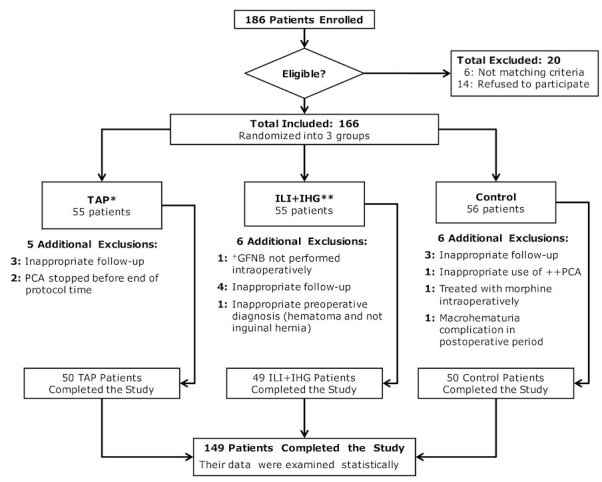
CONSORT Flow Chart. ^*^TAP group, transversus abdominis plane block group; ^**^ILI+IHG, ilioinguinal and iliohypogastric nerve blocks group; +GFNB, genitofemoral nerve block; ++PCA, patient-controlled analgesia with morphine.

Based on the exclusion criteria, 20 patients were excluded from the study. The inclusion group (166 adult male patients) was randomized into three groups: TAP, ILI+IHG, and control (Figure 1). Randomization was done using a computer-generated table of random numbers, placing them in a sealed envelope, and then opening the envelope on the morning of surgery. Patients in the control group did not undergo a pre-op peripheral nerve block; however, the surgeon intraoperatively blocked the genital branch of the genitofemoral nerve in all patients in all three groups.

The patients and the investigator who collected the data during the post-op period were blinded to the group assignments throughout the trial.

Premedication was limited to intravenous (IV) fentanyl (0.5 μg/kg), midazolam (0.03 mg/kg), and local anesthesia via injection of lidocaine (3–5 mL; 10 mg/mL). Standard American Society of Anesthesiologists monitoring and supplemental oxygen (40% via mask; 5 L/min) were applied throughout the procedure.

The same anesthesiologists (A. Stav or L.R.) performed all pre-op US-guided peripheral nerve blocks using an ultrasound system (SonoSite S-Nerve, SonoSite, Bothell, WA, USA) with a 6–13 MHz linear array transducer (L25x). A 22-gauge 80 mm Pajunk Sono Tap cannula (Pajunk® Medical Produkte GmbH, Geisingen, Germany) was used to perform the blocks. All pre-op blocks were performed using aseptic techniques in the “block room.”

The local anesthetic injected for the nerve blocks comprised bupivacaine 5 mg/mL with adrenaline 5 μg/mL. The same volume of 20 mL of local anesthetic was injected for TAP and ILI+IHG to ensure similar local anesthetic doses and volumes, and to facilitate appropriate comparison of perioperative pain and morphine consumption between the groups.

The TAP and ILI+IHG procedures were conducted using techniques described by Hadzic.^16^

The genital branch of the genitofemoral nerve was blocked intraoperatively in all patients in all three groups by the surgeon during exploration of the spermatic cord under direct vision at the level of the internal inguinal ring. “All the genital branches passed through the ventral aspect of the internal ring, entered the ring and continued within the spermatic cord.”^17^ The block was induced by injecting 10 mL of bupivacaine 5 mg/mL without adrenaline.

The success of the sensory block was tested by pin-prick in the inguinal crease (from the projection of the internal inguinal ring and medial up to the projection of the external ring) 30 min after completion of the pre-op procedure (both groups) by an independent investigator who was blinded to the previously performed block. Patients were additionally excluded from the study if a failed block was diagnosed (normal sensation).

A standardized general anesthesia protocol was used for all patients. Lornoxicam 8 mg IV was injected 30 min before the end of surgery.

All operations were performed by or under the supervision of the same experienced surgeons (A.T. or M.K.). Morphine was initiated via PCA IV in the postanesthesia care unit (PACU) for all patients. The PCA protocol was as follows: a loading bolus of 2 mg IV morphine; 1 mg for the subsequent bolus, followed by a lockout period of 5 min, with a maximal dosage of 50 mg within a 4-hour period. Patient-controlled analgesia continued post-op for 24 hours.

Variables assessed before and during surgery included patient characteristics (age, height, and body weight (BW)). Body mass index (BMI) was calculated using the following formula: BMI = BW (kg)/height^2^ (m^2^). Preoperative pain at rest and during motion was measured using a visual analogue scale (VAS).^18^ If the oxygen saturation decreased during surgery, or bradycardia, low blood pressure, or other events persisted for more than 2 min, the patient was excluded from the study. The aim of this exclusion was to remove potential temporary intraoperative brain tissue oxygen desaturation, which may influence mental state^19^ and pain intensity estimation during the post-op period when assessed by VAS. Pain intensity in the PACU was measured using a modified nurses’ assessment of postoperative pain scale (NP):^20^ 0=no pain, or the patient is asleep; 1=mild pain; 2=moderate pain; 3=severe pain; 4=intolerable pain.

If moderate, severe, or intolerable pain was experienced by the patient in the PACU immediately post-op, then morphine was initially injected using a titration method until pain relief was achieved,^21^,^22^ followed by PCA. Morphine consumption in the PACU, post-op nausea and vomiting (PONV) (yes/no), and time of stay were assessed and statistically compared between the groups. In addition, data were collected after discharge from the PACU, i.e. during post-op day 0 regarding pain intensity at rest and during motion (measured by VAS), morphine consumption after PACU discharge and up to 24 hours post-op, and other analgesic consumption (oral dipyrone (metamizole), IV or oral paracetamol (acetaminophen), or non-steroidal anti-inflammatory drugs). The investigator collecting the data 24 hours after PACU discharge was blinded regarding the type of previously performed block.

The primary end-point of the study included three parameters: pain intensity immediately post-op, morphine and other analgesic consumption 24 hours post-op, and pain intensity following termination of the effect of the peripheral nerve blocks in the “block” groups, i.e. 24 hours post-op.

The parameters for the secondary end-point were: PONV (yes/no), PACU time of stay, and the correlation between block performance time and the patients’ body mass index (BMI).

### Statistical Analysis

*A priori* power analysis was performed using “G^*^ Power 3.0.10”© with a fixed effects, omnibus, one-way ANOVA for all groups. A total sample size of 66 was considered adequate to achieve an effect size of 0.5 with an α error probability of 0.05 and a power (1-β error probability) of 0.95. After all exclusions, our study included 149 patients.

Statistical analysis was performed using “IBM SPSS Statistics 20”©. Continuous numerical parameters were analyzed according to the Shapiro–Wilk test for distribution normality, followed by the Levene test for homogeneity of the variances (if a normal distribution was determined). Parameters with a normal distribution and homogeneous variances were compared by one-way ANOVA followed by Tukey’s *post-hoc* test, if necessary.

The Kruskal–Wallis test was used when an abnormal distribution of the continuous variables was detected. The Mann–Whitney *post-hoc* test was used following the Kruskal–Wallis test, if necessary.

The intensity of VAS-measured pain was also analyzed using the Kruskal–Wallis test followed by the Mann–Whitney test, if necessary.

Frequency tables and Pearson chi-square tests were used to compare the proportions between the categorical variables among the groups. Pearson correlation was used to assess the correlation between BMI and block performance time in each group. A value of *P*<0.05 was considered statistically significant.

## RESULTS

Study data were collected between October, 2011 and September, 2015. The results of our trial are presented according to the standards of reporting Clinical Trials (CONSORT) statement using a CONSORT flow chart (Figure 1). The data obtained from 149 patients were statistically analyzed until the end of the study. The pre-op variables were comparable among the groups (Table 1). There were no differences in surgery duration, and there was no correlation between the block performance time and BMI. There were no complications during or immediately after the blocks in the TAP and ILI+IHG groups.

**Table 1:** Preoperative Data.

	TAP (*n*=50)	ILI+IHG Group (*n*=49)	Control Group (*n*=50)	*P*
Age (years)	50±17	46±19	49±16	NS
Height (m)	1.75±0.07	1.73±0.07	1.73±0.08	NS
BW (kg)	77.83±12.15	77.24±10.79	74.38±11.92	NS
BMI	25.47±3.55	25.84±3.67	24.66±3.44	NS
Pain* (rest) in mm	6.54±12.84	9.12±19.68	5.34±13.56	NS
Pain* (motion) in mm	39.66±27.51	28.61±29.17	29.60±32.10	NS
Block performance time (min)	11.48±4.82	11.80±4.07		NS

All values are presented as mean±SD.

* Pain intensity at rest and during motion were measured by visual analogue scale (VAS) in mm.

BMI, body mass index; ILI+IHG, ilioinguinal and iliohypogastric nerve blocks group; NS, no statistically significant difference between groups; TAP, transversus abdominis plane block group.

Pain intensity in the PACU and morphine consumption was not significantly different between the TAP and ILI+IHG groups; however, they were significantly decreased when compared with the control group (Table 2). This was one parameter of the primary end-point of our study.

**Table 2:** Pain Intensity and Drug Consumption.

	TAP (*n*=50)	ILI+IHG (*n*=49)	CG (*n*=50)	*P* value		
				TAP versus ILI+IHG	TAP versus CG	ILI+IHG versus CG
Pain intensity (by NP scale) in PACU	0.72	0.86	1.6	0.373	0.001	0.008
Morphine consumption (mg) in PACU	4.24	3.94	7.08	0.573	0.049	0.017
Pain at rest, 24 h post operation by visual analogue scale (mm, 1–100)	37.46	30.84	35.28	NS	NS	NS
Pain at motion, 24 h post operation by visual analogue scale (mm, 1–100)	46.37	39.87	51.21	NS	NS	NS
Dipyrone consumption during 24 h after operation	327	41	380	<0.0001	0.37	<0.0001
Morphine consumption, first day after PACU discharge	18.76	10.69	18.12	0.004	0.901	0.001

Control group: Patients in this group received intraoperative genitofemoral block only.

CG, control group; ILI+IHG, Ilioinguinal and iliohypogastric nerve blocks group; NP, modified nurses’ assessment of postoperative pain scale; PACU, post anesthesia care unit; TAP, transversus abdominis plane block group. See text for details.

Prophylaxis of PONV with 8 mg ondansetron IV prior to induction of anesthesia was effective. No PONV occurred in the 147 evaluated patients. One patient in the TAP group and one patient in the control group suffered from nausea, but no vomiting occurred during the immediate post-op period or during the 24 hours post-op. This was the secondary end-point of the trial.

The duration of PACU stay was similar in all groups.

Following PACU discharge, the patients in all groups were treated with PCA.

Pain intensity (VAS) at rest and during motion 24 hours post-op was similar in all groups (Table 2). Morphine and dipyrone consumption following PACU discharge and up to 24 hours post-op were significantly decreased in the ILI+IHG group compared with the other two groups. There were no differences between the TAP and the control group (Table 2). Analgesic consumption during the 24 hours post-op was another parameter of the primary end-point of our trial.

## DISCUSSION

Acute pain following open hernia repair is maximal on the day of surgery^23^ (the Lichtenstein mesh repair method was included in another trial of Callesen et al.^24^). Both regional anesthesia methods (TAP and ILI+IHG) have been previously recommended as components of multimodal analgesia in the post-op period to induce a significant reduction in pain during the initial 24 hours following open hernia repair.^2^–^6^ This raises the question as to which block should be used in specific cases.

In contrast to Aveline et al.,^8^ comparing US-guided TAP with landmark-guided ILI+IHG, we compared post-op pain relief and morphine consumption immediately after surgery and during the 24 hours post-op between two US-guided techniques: posterior TAP versus ILI+IHG. The TAP and ILI+IHG approaches represent a significant difference between investigations, with subsequent differences in the results because visualization of target structures enabled the possibility of accurately injecting the local anesthetic into the transversus abdominis plane (in TAP group) or around the ilioinguinal and iliohypogastric nerves (ILI+IHG group). Sasaoka et al.^15^ in 2005 and Asad et al. in 2009^25^ have indicated that the addition of a genitofemoral nerve block (genital branch of the genitofemoral nerve) to ILI+IHG provides hemodynamic stability during sac traction in children following hernia repair, i.e. a combination of the three nerve blocks provides better pain relief compared with ILI+IHG alone. For appropriate comparisons of the post-op pain relief and morphine consumption, we blocked the genital branch of the genitofemoral nerve in all patients in all groups. This approach enables neutralization of painful impulses originating from structures innervated by the genital branch of the genitofemoral nerve. This is a significant strength of our trial and a critical difference between our investigation and that of Aveline et al.^8^

Our inclusion and exclusion criteria enabled the selection and comparison of similar groups of patients with regard to the pre-op data, gender (all patients were men), and patients suffering from primary inguinal hernia (but not from recurrent, sliding, or inguinoscrotal huge hernia). Primary open hernioplasty via the Lichtenstein tension-free technique was used in all patients. The same concentration and volume of local anesthetic was used for all patients in the two pre-op block groups. All pre-op blocks were performed by the same experienced anesthesiologists; an intraoperative block of the genital branch of the genitofemoral nerve was performed or controlled by the same experienced surgeons. A very high level of similarity among all three groups is a strong advantage of our trial as compared to other similar studies.^8^–^10^,^13^,^15^,^25^

In our prospective randomized controlled and observer-blinded trial, pain intensity and morphine consumption immediately post-op (in the PACU) were significantly less in the TAP and ILI+IHG groups compared with the control group; however, there were no differences between the TAP and ILI+IHG groups themselves. This finding indicates that both methods of US-guided blocks produce significant post-op pain relief immediately after surgery, with no significant difference between the procedures.

Twenty-four hours post-op, when the action of bupivacaine has decreased significantly or has ceased, no differences in pain intensity were observed among the all groups. Total morphine and dipyrone consumption in the ILI+IHG group was significantly decreased compared with the TAP and control groups; however, the TAP and control groups were similar. This finding indicates that the ILI+IHG produces superior pain relief compared to TAP during the 24 hours post-op, but not immediately after surgery.

Our results may be explained by the hypothesis that the spread of local anesthetic during and after injection differs following TAP and ILI+IHG. Posterior TAP is a compartment block, and the spread of local anesthetic is relatively large, i.e. from Th7 to L1.^5^,^26^ Following ILI+IHG, the local anesthetic only spreads around the target nerves. We compared the groups with the same injected volume and concentration of bupivacaine (20 mL of 0.5%); thus, we can assume that following ILI+IHG a relatively high quantity of bupivacaine had spread around the target of the two nerves compared with the TAP block. It is possible that the quality of the compared blocks is similar immediately post-op (in the PACU); however, when the quantity of the local anesthetic around and in the nerves decreases, the quality of the blocks changes, i.e. the quality of the ILI+IHG improves. This is one potential explanation for the difference in morphine consumption over 24 hours between the TAP and ILI+IHG groups.

Our study has several limitations as follows:

- There were strong inclusion criteria: limited to men, primary inguinal hernia, and hernioplasty using the Lichtenstein tension-free method. Thus, the conclusions of the trial are relevant for a specific pathology, gender, and method of operation.
- We used two scales for the pain intensity measurement, including the modified NP scale^20^ and the VAS.^18^ Readers should note this difference in the presented results.
- Lornoxicam 8 mg IV was injected 30 min before the hernioplasty ended; dipyrone was used as needed to treat mild pain or discomfort during the post-op period; those agents may have influenced pain intensity and morphine consumption.
- It is impossible to examine the individual anatomical variations of the ilioinguinal and iliohypogastric nerves pre-op. These potential variations have been examined and described by Klaassen et al.^27^ In this work the authors included the observation of the spinal nerve contribution, as well as the communications with other nerves (such as the accessory nerve, subcostal nerves, and lateral femoral cutaneous nerve). Anatomical variations may explain the partial sensory block following partial analgesia in the evaluated patients after both the TAP and ILI+IHG nerve block, as well as after surgery.

## CONCLUSIONS

The data from this prospective, randomized, controlled, and observer-blinded clinical study lead to several important conclusions.

- Both regional anesthesia methodologies (US-guided TAP and US-guided ILI+IHG) may be used as components of multimodal anesthesia following a primary unilateral open Lichtenstein patch tension-free inguinal hernioplasty in adult men.
- Both blocks provided comparative analgesia, as well as a similar morphine-sparing effect immediately after the surgery, which was performed under general anesthesia.
- During the 24 hours after surgery, US-guided ILI+IHG (a truncal block) was superior to US-guided TAP (a compartment block) in terms of the morphine-sparing effect, i.e. ILI+IHG provided significantly better analgesia.
- Prophylaxis of PONV with 8 mg ondansetron IV prior to anesthesia induction was effective.
- The duration of PACU stay was similar in all three groups.
- There was no correlation between the time of block performance and BMI.
- Potential anatomical variations in the ilioinguinal and iliohypogastric nerves, which are impossible to diagnose prior to surgery, could play a role in the intensity of post-op acute pain. This was a significant limitation of the trial.

## Abbreviations

BMI: body mass index
BW: body weight
ILI+IHG: ilioinguinal and iliohypogastric nerve block
IV: intravenously
NP scale: nurses’ assessment of postoperative pain scale
PACU: postanesthesia care unit
PCA: patient-controlled analgesia with intravenous morphine
PONV: post-op nausea and vomiting
pre-op: preoperative or preoperatively
post-op: postoperative or postoperatively
PROSPECT: procedure-specific post-op pain management
TAP: posterior transversus abdominis plane block
US: ultrasound
